# Hypersensitivity Induced by Activation of Spinal Cord PAR2 Receptors Is Partially Mediated by TRPV1 Receptors

**DOI:** 10.1371/journal.pone.0163991

**Published:** 2016-10-18

**Authors:** Petra Mrozkova, Diana Spicarova, Jiri Palecek

**Affiliations:** Department of Functional Morphology, Institute of Physiology, The Czech Academy of Sciences, Prague, Czech Republic; University of Texas Medical Branch at Galveston, UNITED STATES

## Abstract

Protease-activated receptors 2 (PAR2) and transient receptor potential vanilloid 1 (TRPV1) receptors in the peripheral nerve endings are implicated in the development of increased sensitivity to mechanical and thermal stimuli, especially during inflammatory states. Both PAR2 and TRPV1 receptors are co-expressed in nociceptive dorsal root ganglion (DRG) neurons on their peripheral endings and also on presynaptic endings in the spinal cord dorsal horn. However, the modulation of nociceptive synaptic transmission in the superficial dorsal horn after activation of PAR2 and their functional coupling with TRPV1 is not clear. To investigate the role of spinal PAR2 activation on nociceptive modulation, intrathecal drug application was used in behavioural experiments and patch-clamp recordings of spontaneous, miniature and dorsal root stimulation-evoked excitatory postsynaptic currents (sEPSCs, mEPSCs, eEPSCs) were performed on superficial dorsal horn neurons in acute rat spinal cord slices. Intrathecal application of PAR2 activating peptide SLIGKV-NH_2_ induced thermal hyperalgesia, which was prevented by pretreatment with TRPV1 antagonist SB 366791 and was reduced by protein kinases inhibitor staurosporine. Patch-clamp experiments revealed robust decrease of mEPSC frequency (62.8 ± 4.9%), increase of sEPSC frequency (127.0 ± 5.9%) and eEPSC amplitude (126.9 ± 12.0%) in dorsal horn neurons after acute SLIGKV-NH_2_ application. All these EPSC changes, induced by PAR2 activation, were prevented by SB 366791 and staurosporine pretreatment. Our results demonstrate an important role of spinal PAR2 receptors in modulation of nociceptive transmission in the spinal cord dorsal horn at least partially mediated by activation of presynaptic TRPV1 receptors. The functional coupling between the PAR2 and TRPV1 receptors on the central branches of DRG neurons may be important especially during different pathological states when it may enhance pain perception.

## Introduction

Protease-activated receptor 2 (PAR2) belongs to a family of four (PAR1-4) G-protein-coupled receptors (GPCRs) that share a unique mechanism of activation by extracellular and membrane-tethered proteases [1–3]. PARs are cleaved and activated by proteases, generated and released from cells of immune and nervous systems during injury and inflammation [1]. Proteases such as trypsin, mast cell tryptase or serine protease 1 cleave the specific sites of PAR2 extracellular N-terminus to reveal the tethered ligand and activate the receptor [4,5]. PAR2 are present in many tissues like intestine, lungs, kidneys, endothelium, mast cells and in the central and peripheral nervous systems in neurons and astrocytes [5–8]. PAR2 in the peripheral and central nervous system are involved in neuronal and astrocytic survival, proliferation, release of neuropeptides and also modulate the function and activity of ion channels [9]. In addition, PAR2 are important players in response to tissue injury, protease-driven inflammation, nociception and also in tissue repair [7,10].

The expression of PAR2 was documented throughout the nervous system, in the brain, spinal cord and dorsal root ganglia (DRG), [11,12]. A large number (> 60%) of DRG neurons that express PAR2 were identified primarily as small-sized neurons, with some medium- to large-sized neurons [11,13,14]. There is mainly functional electrophysiological evidence for the presence of PAR2 in the spinal cord dorsal horn [15–17], while recently PAR2 were detected also by western blot analysis of the rat spinal cord tissue [18].

Several intracellular pathways, involving activation of phospholipases and protein kinases (PKs), are linked downstream to the PAR2 activation. One important signalling cascade, implicated in nociception, involves activation of phospholipase C (PLC) and generation of inositol trisphosphate (IP_3_), leading to mobilization of intracellular Ca^2+^ and activation of second messenger PKC, while other key protein kinases (PKA, PKD) may be also activated [13,19–22]. The increase of intracellular Ca^2+^ concentration initiates many signalling events, including activation of the phospholipase A_2_-cyclooxygenase cascade [23]. It was demonstrated that intrathecal administration of PAR2 agonist induced cyclooxygenase activation and PGE2 release in the spinal cord tissue [24].

Activation of PAR2 indirectly modulates function of some transient receptor potential (TRP) ion channels, important for nociceptive signalling. Sensitization of TRPV1, TRPV4 and TRPA1 receptors was demonstrated after PAR2 activation [13,14,19,25,26]. TRPV1 (vanilloid 1) is a non-selective cation channel that integrates nociceptive stimuli in the periphery and at the spinal cord level and plays a critical role in the processing of somatic and visceral pain [27–31]. TRPV1 receptors are highly expressed in small-diameter DRG neurons and may be directly activated by different exogenous and endogenous stimuli [32,33]. The majority of TRPV1 expressing DRG neurons (almost 90%) co-express PAR2 [13,14]. In DRG neurons, PAR2-induced TRPV1 sensitization involves activation of PLC [13], PKC and PKA [34]. Sensitized TRPV1 receptors may be subsequently activated by low concentration of endogenous agonists [29,35]. In addition, PAR2 activation evoked [11] and enhanced capsaicin (TRPV1 agonist) stimulated release of pronociceptive neuropeptides, substance P (SP) and calcitonin gene-related peptide (CGRP), within the spinal cord dorsal horn [13]. It was also demonstrated that increased TRPV1 expression in the superficial dorsal horn under pathological conditions was dependent on PAR2 activation [18,36,37].

Proteases activating PAR2 have widespread proinflammatory effects, partially via neurogenic mechanism [11,38,39]. Activation of PAR2 on the peripheral nerve endings leads to sensitization of DRG neurons and stimulate release of SP and CGRP in the peripheral tissues and in the spinal cord [11,40,41]. PAR2-induced increase of cytosolic Ca^2+^ concentration was shown not only in neurons [11], but also in astrocytes [42,43]. Intraplantar injection of PAR2 agonist induced persistent thermal hyperalgesia, which was prevented by TRPV1 receptors blockade or deletion [13,14]. Peripheral injection of low, subinflammatory doses of PAR2 agonist also induced thermal and mechanical hyperalgesia and elevated Fos protein expression in the spinal cord [40]. Thermal hyperalgesia induced by intrathecal administration of PAR2 agonist, mediated by activation of cyclooxygenase 1 and 2 was also documented [24]. In addition, activation of PAR2 is involved in several pathological pain states as was demonstrated in inflammatory [4], bone cancer [36], chemotherapeutic agent-induced pain [18] or osteoarthritis [44]. These results indicate an important role of PAR2 in peripheral inflammatory pain and suggest their involvement in nociceptive transmission at spinal cord level.

The synthetic peptide corresponding to the tethered ligand domain, SLIGKV-NH_2_, mimics the effects of endogenous activators. In our experiments, we investigated the role of spinal cord PAR2 activation in nociceptive modulation using administration of this activating peptide *in vivo* and *in vitro*. Patch-clamp recordings from lamina I and II_(outer)_ dorsal horn neurons in spinal cord slices were used to study the effect of PAR2 activation on the properties of miniature, spontaneous and dorsal root stimulation-evoked excitatory postsynaptic currents (mEPSC, sEPSC, eEPSC). Intrathecal administration of SLIGKV-NH_2_ was used to study the behavioural changes in the responsiveness to thermal and mechanical stimuli. Specific antagonists were used to evaluate the involvement of TRPV1 receptors and protein kinases after the PAR2-induced modulatory effects.

## Materials and Methods

### Ethics Statement

All experiments were approved by the Animal Care and Use Committee of the Institute of Physiology CAS and were carried out in accordance with the guidelines of the International Association for the Study of Pain, the U.K. Animals (Scientific Procedures) Act, 1986 and associated guidelines, and EU Directive 2010/63/EU for animal experiments. All efforts were made to minimize animal suffering, to reduce the number of animals used, and to utilise alternatives to in vivo techniques, if available.

### Animal care and utilization

Altogether 71 male Wistar rats (Institute of Physiology, CAS) were used in this study. The animals were housed in a temperature-controlled facility at 23 ± 2°C with free access to food and water and maintained on a 12 h light, 12 h dark cycle and were checked twice a day. All the animals were handled only for a necessary period of time and throughout the experiment did not show any signs of stress or illness. Animals were sacrificed at the end of the experiment by deep anaesthesia with ketamine (150 mg/kg) and xylazine (20 mg/kg), subsequent medulla interruption and exsanguination. No animal was excluded from the study or sacrificed for disease.

### Spinal cord slice preparation

Acute spinal cord slices were prepared from male Wistar rats on postnatal days P21–P23, similar to previously published data [35]. After deep anaesthesia with 4% isoflurane (Forane^®^, Abbott), the lumbar spinal cord was removed and immersed in oxygenated ice-cold dissection solution containing (in mM): 95 NaCl, 1.8 KCl, 7 MgSO_4_, 0.5 CaCl_2_, 1.2 KH_2_PO_4_, 26 NaHCO_3_, 25 D-glucose, 50 sucrose. The spinal cord was then fixed to vibratome stage (Leica VT1200S, Germany) using cyanoacrylate glue in a groove between two agar blocks. Transverse slices 300 μm thick were cut from the lumbar segment L3–L5, incubated in the dissection solution for 30 min at 33°C and then stored in a recording solution at room temperature until used for the electrophysiological experiments. The recording solution contained (in mM): 127 NaCl, 1.8 KCl, 1.2 KH_2_PO_4_, 2.4 CaCl_2_, 1.3 MgSO_4_, 26 NaHCO_3_, 25 D-glucose. For the actual measurement, slices were transferred into a recording chamber continuously perfused with the recording solution at a rate ~ 2 ml/min. All extracellular solutions were saturated with carbogen (95% O_2_, 5% CO_2_) during the whole process.

### Patch-clamp recordings

Patch-clamp recordings were made in acute spinal cord slices from superficial dorsal horn neurons (laminae I and II_outer_). Individual neurons were visualized using a differential interference contrast (DIC) microscope (Leica, DM LFSA, Germany) equipped with an near infrared-sensitive camera (Hitachi KP-200P, Japan) with a standard TV/video monitor. Patch pipettes were pulled from borosilicate glass tubing with resistances of 3.5–6.0 MΩ when filled with intracellular solution. The intracellular pipette solution contained (in mM): 125 gluconic acid lactone, 15 CsCl, 10 EGTA, 10 HEPES, 1 CaCl_2_, 2 MgATP, 0.5 NaGTP and was adjusted to pH 7.2 with CsOH. Voltage-clamp recordings in the whole-cell configuration were performed with an Axopatch 200B amplifier and Digidata 1440A digitizer (Molecular Devices, USA) at room temperature (~ 23°C). Whole-cell recordings were low-pass filtered at 2 kHz and digitally sampled at 10 kHz. The series resistance of neurons was routinely compensated by 80% and was monitored during whole experiment. AMPA receptor-mediated spontaneous, miniature and evoked EPSCs were recorded from neurons clamped at -70 mV in the presence of 10 μM bicuculline and 5 μM strychnine. Miniature EPSCs were distinguished by the addition of 0.5 μM tetrodotoxin (TTX) to the bath solution. In order to record evoked EPSCs, a dorsal root was stimulated using a suction electrode with glass pipette filled with an extracellular solution using a constant current isolated stimulator (Digitimer DS3, England). The intensity of the stimulation was adjusted to evoke stable EPSC with 0.5 ms stimulus duration and at least 3× the minimal stimulus current at a frequency of 0.033 Hz.

The experiments started with control recordings (4 min), followed by PAR2 agonist (SLIGKV-NH_2_, 100 μM, 4 min) application. In the groups where antagonist was used (SB 366791, 10 μM; staurosporine, 250 nM), it was applied for 4 min after the control recording as a pre-treatment and then with SLIGKV-NH_2_ (100 μM) as co-application. Concentration of SB 366791 used for experiments was determined from IC_50_ = 7.5 ± 1.8 nM [45] and our earlier studies [35,46], concentration of SLIGKV-NH_2_ was based on the EC_50_ (~ 1 μM) [47] and previously used effective concentrations [17]. The mEPSC and sEPSC activity was always evaluated during the last two minutes of the specific application. The evoked EPSCs were recorded every 30 s, the average amplitude of 4 evoked currents in the last two minutes of the particular application was always used for evaluation of the specific condition. Neurons with capsaicin-sensitive afferent input were identified by an increase of EPSC frequency (> 20%), measured after capsaicin (200 nM) application at the end of each recording protocol.

Software package pCLAMP 10 (Molecular devices, USA) was used for data acquisition and subsequent off-line analysis. Data segments of 2 min duration were analysed for each experimental condition. Only EPSCs with an amplitude of 5 pA or greater (which corresponded to at least twice the recording noise level) were included in the frequency analysis. The same events and data segments were used for amplitude analysis. Data are expressed as mean ± standard error of the mean (SEM). Data were normalized as a percentage of the control value (100%). For statistical analysis of significant differences One Way ANOVA or One Way repeated measures ANOVA were used followed by Holm-Sidak post hoc test. A Kolmogorov-Smirnov test was used to evaluate statistical significance for cumulative data.

### Drug treatment

All basic chemicals, used for the preparation of the dissection, recording and intracellular solution, were of analytical grade and purchased from Sigma-Aldrich (Prague, Czech Republic) and Tocris Bioscience (Bristol, UK). Capsaicin, SLIGKV-NH_2_, VKGILS-NH_2_, SB 366791 and staurosporine were dissolved in DMSO, which had a concentration of < 0.1% in the final solution.

### Intrathecal catheter implantation

Experiments were conducted using adult male Wistar rats (250–300 g). Lumbosacral catheters were implanted between the L4–L5 vertebrae one week before the experiment. Catheter implantations were performed under brief isoflurane (3%, Forane^®^, Abbott), followed by ketamine (100 mg/kg) and xylazine (16 mg/kg) anaesthesia. The catheters were constructed from polyethylene tubing (PE5) and were fixed with dental cement (Duracryl) to the vertebral bones. The other end of each catheter was fixed to PE10 tubing and externalized on the back of the animal. The positions of the catheters were verified by a dye injection at the end of each experiment. Intrathecal drugs were applied and flushed (45 or 50 μl) from the catheter by physiological solution: SLIGKV-NH_2_ and VKGILS-NH_2_ (10 μl, 8 μg), SB 366791 (15 μl, 0.43 μg), staurosporine (15 μl, 0.014 μg).

### Behavioural tests

Experiments were conducted on rats, previously implanted with intrathecal catheter, kept in plastic cages with soft bedding, with free access to food and water and maintained on a 12 h light, 12 h dark cycle. The paw withdrawal latency (PWL) to thermal stimulation was tested using a plantar test apparatus (Ugo Basile, Italy) with radiant heat applied to the plantar surface of each hindpaw. Rats were placed in nonbinding, clear plastic cages on a clear glass plate, elevated to allow application of controlled heat source underneath. Each rat was left to adapt to the testing environment for at least 15 min prior to any stimulation. The hindpaw withdrawal latencies were measured automatically with the apparatus. Each hindpaw was tested 4 times with at least 5 min between the trials. Baseline withdrawal latencies were determined in all animals before any experimental procedure.

The paw withdrawal threshold (PWT) to tactile stimulation was tested manually with an electronic von Frey device (IITC Life Science, Model 2390 Series) where a probe tip was applied to the plantar surface of each hindpaw. The PWT was defined as the force (mN) that evoked an active paw withdrawal response. Each paw was tested 4 times at each time interval and the mean was calculated. The averaged values from the left and right hind paws in individual animals were then averaged in the experimental groups. All data are expressed as mean ± SEM. One Way repeated measure ANOVA with Bonferroni post hoc test and Two-Way ANOVA with Student Newman Keuls test was used to identify statistically significant differences.

## Results

### Activation of spinal PAR2 in thermal and mechanical sensitivity

**Fig 1:**
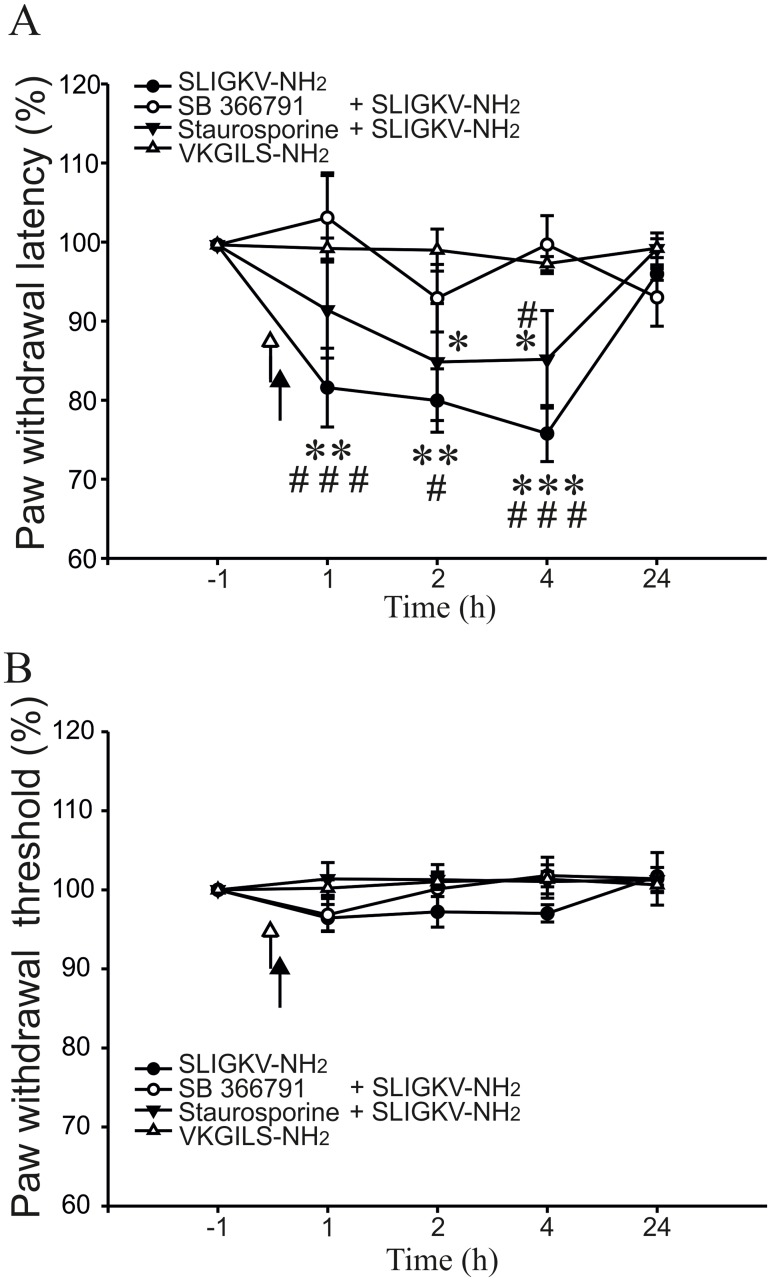
Activation of spinal PAR2 induced thermal hyperalgesia. **(A)** Intrathecal administration of PAR2 activating peptide SLIGKV-NH_2_ (8 μg, 10 μl, n = 7) decreased the PWLs to radiant heat stimulation for several hours after the treatment. An inactive reverse peptide VKGILS-NH_2_ (8 μg, 10 μl, n = 6) did not change the thermal threshold. TRPV1 antagonist SB 366791 (0.43 μg, 15 μl, n = 6) pre-treatment prevented SLIGKV-NH_2_ induced decrease of PWLs. Staurosporine (0.014 μg, 15 μl, n = 7) pre-treatment also partially blocked the PWL decrease induced by SLIGKV-NH_2_. White arrowhead: application of SB 366791 or staurosporine. Black arrowhead: application of SLIGKV-NH_2_ or VKGILS-NH_2_ **(B)** Paw withdrawal threshold to mechanical stimulation with von Frey filament was not significantly affected by any of the intrathecal treatments. Statistical differences between groups with various treatments were identified using Two-way ANOVA followed by multiple comparisons Student Newman Keuls test (*p < 0.05, **p < 0.01, ***p < 0.001 versus inactive peptide VKGILS-NH_2_; ^#^p < 0.05, ^###^p < 0.001 versus SB 366791 + SLIGKV-NH_2_).

The role of spinal PAR2 receptors in thermal and mechanical hypersensitivity was investigated in behavioural experiments. The concentration of PAR2 activating peptide SLIGKV-NH_2_, was based on effective doses used in previous studies [15,17]. Intrathecal administration of SLIGKV-NH_2_ (8 μg in 10 μl of saline) decreased the paw withdrawal latency in response to a thermal stimulus already one hour after the treatment (81.6 ± 4.9%, n = 7, p < 0.001, Fig 1A). This decrease of PWLs was present at 2 h (80.3 ± 4.1%, p < 0.001) and was even more pronounced at 4 h after the SLIGKV-NH_2_ administration (76.1 ± 3.5%, p < 0.001) compared to control pre-treatment values. The PWLs returned close to the control values at 24 h after the SLIGKV-NH_2_ administration (96.3 ± 0.8%).

In control experiments an inactive reverse peptide VKGILS-NH_2_ was used. Intrathecal administration of VKGILS-NH_2_ (8 μg in10 μl of saline) did not change the PWL at any of the tested time points (1 h, 99.5 ± 1.3%; 2 h, 99.3 ± 2.6%; 4 h, 97.6 ± 1.8%; 24 h, 99.6 ± 1.1%; n = 6, Fig 1A).

Another set of behavioural experiments was performed to test the role of spinal TRPV1 receptors in hyperalgesia induced by spinal PAR2 activation. Intrathecal administration of TRPV1 antagonist SB 366791 (0.43 μg in 15 μl of saline, n = 6) 5 min before the SLIGKV-NH_2_ (8 μg in 10 μl of saline) treatment prevented any significant change from the control values (1 h, 103.4 ± 5.3%; 2 h, 93.2 ± 4.3%; 4 h, 100.0 ± 3.6%; 24 h, 93.4 ± 3.6%, Fig 1A). Pre-treatment with SB 366791 thus completely abolished the thermal hyperalgesia induced by the SLIGKV-NH_2_ application alone.

The involvement of PKs activation in PAR2-induced hyperalgesia was investigated next. A broad spectrum PKs inhibitor staurosporine (0.014 μg in 15 μl of saline, n = 7) was administered 5 min before SLIGKV-NH_2_ (8 μg in 10 μl of saline). Paw withdrawal latencies were slightly decreased after this treatment, while only at 2 h and at 4 h intervals it reached a statistical significance (1 h, 91.8 ± 6.1%; 2 h, 85.2 ± 7.4%, p < 0.05; 4 h, 85.5 ± 6.2%, p < 0.05; 24 h, 99.5 ± 2.0%, Fig 1A). Our results indicate that inhibition of spinal PKs significantly attenuated the PAR2-induced thermal hyperalgesia.

Tests of mechanical sensitivity, performed at the same time, did not show any effect after i.t. application of any of the tested drugs (SLIGKV-NH_2_, VKGILS-NH_2_, SB 366791, staurosporine, Fig 1B). These results suggest that activation of spinal PAR2 failed to change mechanical sensitivity at any of the tested time points.

### Modulation of mEPSCs in spinal cord slices by PAR2 activation

Modulation of mEPSCs activity recorded from superficial dorsal horn neurons after PAR2 activation was tested *in vitro* using spinal cord slices. Miniature EPSCs were recorded in 41 neurons, where the average control mEPSC frequency was 0.8 ± 0.1 Hz. Out of these 41 neurons 38 showed an increase of mEPSC frequency (7.9 ± 1.8 Hz, n = 38, p < 0.001) after TRPV1 agonist capsaicin (0.2 μM) application at the end of the recording. This suggests the presence of presynaptic TRPV1 receptors in great majority of the recorded neurons.

**Fig 2:**
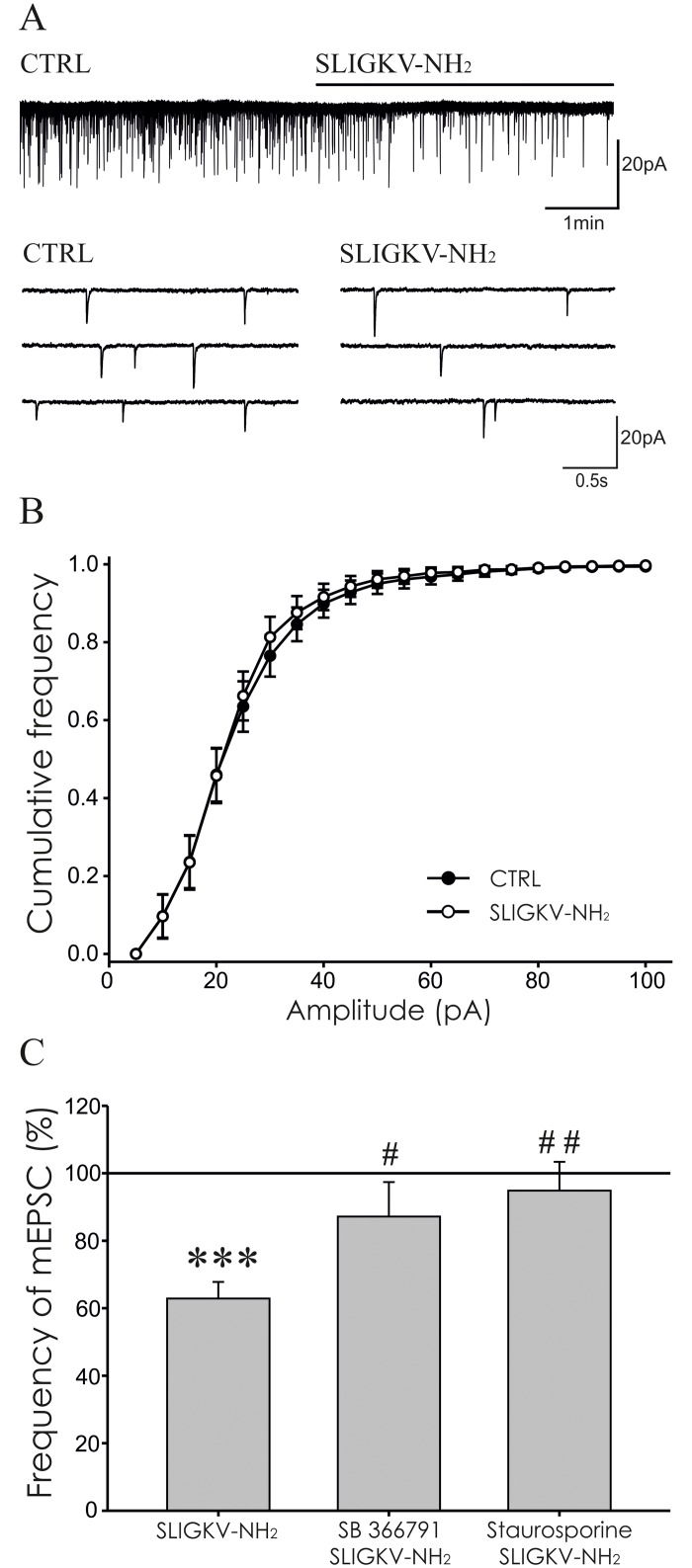
Activation of PAR2 decreased the frequency of mEPSCs. **(A)** Application of SLIGKV-NH_2_ (100 μM, 4 min) lowered the frequency of mEPSC as is documented in the recording from one superficial dorsal horn neuron in acute spinal cord slice. **(B)** Cumulative amplitude analysis of mEPSCs under control conditions and during application of SLIGKV-NH_2_ (100 μM, 4 min, n = 17) did not show statistically significant difference. **(C)** Application of SLIGKV-NH_2_ (100 μM, 4 min) decreased the mEPSC frequency (n = 17; ***p < 0.001) compared to the pretreatment period (100%). Co-application of TRPV1 antagonist SB 366791 (10 μM, 4 min, n = 8) or staurosporine (250 nM, 4 min, n = 10) prevented the inhibitory effect of SLIGKV-NH_2_ (100 μM) treatment and the mean mEPSC values were statistically different compared to the application of SLIGKV-NH_2_ alone (^#^p < 0.05, ^##^p < 0.01).

Application of SLIGKV-NH_2_ (100 μM, 4 min) significantly decreased the mEPSC frequency to 62.8 ± 4.9% (n = 17, p < 0.001), when compared to the pre-treatment values (Fig 2A and 2C). The inhibitory effect on the mEPSC frequency persisted during the 4 minutes washout period (60.7 ± 5.5%, p < 0.001). In a set of control experiments inactive peptide VKGILS-NH_2_ (100 μM) did not elicit any changes of mEPSC frequency (99.3 ± 6.6%, n = 6).

Possible interaction of PAR2 and TRPV1 receptors was evaluated next. Application of TRPV1 antagonist SB 366791 (10 μM, 4 min) did not change the frequency of mEPSC (103.1 ± 8.3%, n = 8). Subsequent co-application of SB 366791 (10 μM) with SLIGKV-NH_2_ (100 μM, 4 min) also did not change the mEPSC frequency significantly (87.2 ± 10.2%, n = 8, Fig 2C), when compared to the period of pre-treatment with SB 366791. These results indicate that application of TRPV1 antagonist prevented the PAR2 activation-induced inhibitory effect on the mEPSC frequency.

Involvement of protein kinases activation in the PAR2-induced inhibitory effect on mEPSC frequency was evaluated in another group of neurons. Application of staurosporine (250 nM, 4 min) alone had no effect on the mEPSC frequency (97.3 ± 15.9%, n = 10). Subsequent co-application of staurosporine (250 nM) with SLIGKV-NH_2_ (100 μM, 4 min) also did not change the mEPSC frequency (94.9 ± 8.5%, n = 10, Fig 2C), when compared to the pretreatment with staurosporine alone.

The mean value of the mEPSC frequency inhibition induced by the PAR2 agonist application alone was significantly different from the changes induced by the combination of PAR2 agonist with TRPV1 and PKs antagonists (Fig 2C). These results show that staurosporine and SB 366791 prevented the PAR2 mediated mEPSC frequency inhibition.

The average amplitude of the control mEPSCs was 22.7 ± 2.4 pA and did not change significantly during the SLIGKV-NH_2_ application (21.7 ± 2.1 pA, n = 17, 100 μM) in the first group of neurons. No change of mEPSC amplitude was also present in the cumulative amplitude analysis (Fig 2B). Likewise there was no change of mEPSC amplitude in any of the other experimental groups (control 21.9 ± 1.5 pA, VKGILS-NH_2_ 21.6 ± 1.3 pA, n = 6; control 22.2 ± 1.7 pA, SB 366791 22.4 ± 2.0 pA, SB 366791/SLIGKV-NH_2_ 21.9 ± 1.6 pA, n = 8; control 22.5 ± 2.5 pA, staurosporine 21.2 ± 2.5 pA, staurosporine/SLIGKV-NH_2_ 20.9 ± 1.8 pA, n = 10).

### Modulation of sEPSCs by PAR2 activation

The effect of PAR2 activating peptide application on spontaneous EPSCs was studied in another group of superficial dorsal horn neurons. In accordance with our previous findings (Spicarova and Palecek, 2009), the basal control sEPSC frequency (1.4 ± 0.1 Hz, n = 42) was significantly higher than the average frequency of mEPSCs recorded in the previous group (0.8 ± 0.1 Hz, n = 41, p < 0.001). Out of these 42 neurons, 38 showed sEPSC frequency increase (6.7 ± 1.9 Hz, n = 38, p < 0.001) after capsaicin (0.2 μM) application at the end of the experiment.

**Fig 3:**
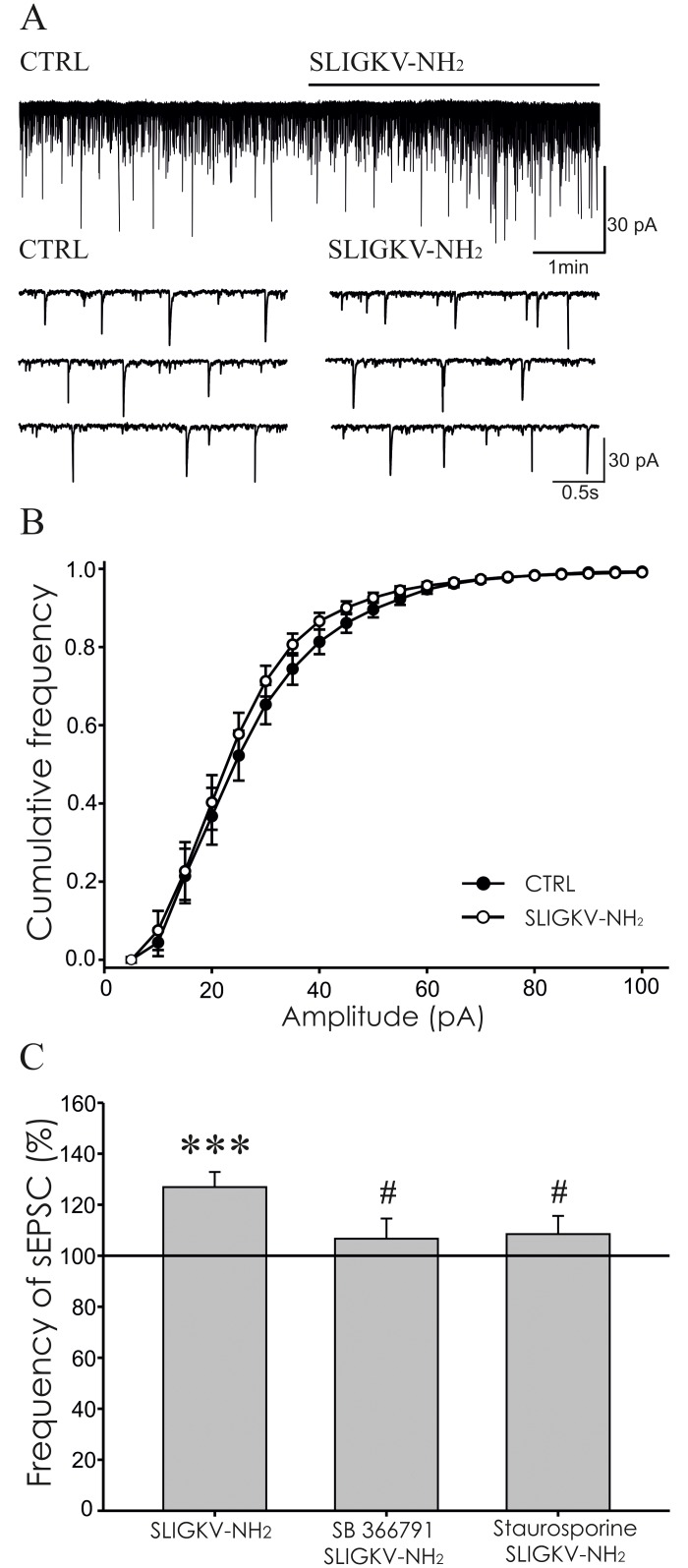
PAR2 activation increased the frequency of sEPSCs. **(A)** Application of SLIGKV-NH_2_ (100 μM, 4 min) increased the sEPSC frequency as documented in recording from one superficial dorsal horn neuron. **(B)** The amplitude of the sEPSCs did not change during SLIGKV-NH_2_ application (100 μM, 4 min, n = 17). **(C)** Application of SLIGKV-NH_2_ (100 μM, 4 min) increased the sEPSC frequency compared to the pre-treatment values set as 100% (n = 17; ***p < 0.001). Application of TRPV1 antagonist SB 366791 (10 μM, 4 min, n = 10) or staurosporine (250 nM, 4 min, n = 9) prevented the excitatory effect of SLIGKV-NH_2_ treatment and the mean sEPSC frequency values were statistically different from the application of SLIGKV-NH_2_ alone (^#^p < 0.05).

Bath application of SLIGKV-NH_2_ (100 μM, 4 min) significantly increased the sEPSCs frequency to 127.1 ± 4.8% (n = 17, p < 0.001), compared to the pre-treatment values (Fig 3A and 3C). The excitatory effect of the SLIGKV-NH_2_ application on the sEPSC frequency slightly diminished, but persisted during the 4 minutes washout period (115.5 ± 7.4%, p < 0.05). In the group of control experiments, inactive peptide VKGILS-NH_2_ (100 μM, 4 min) application did not elicit any change of the sEPSC frequency (94.8 ± 7.9%, n = 6).

Application of SB 366791 (10 μM, 4 min) did not change the sEPSCs frequency (97.5 ± 9.2%, n = 10) in another set of experiments. Subsequent co-application of SB 366791 (10 μM) with SLIGKV-NH_2_ (100 μM, 4 min) similarly did not change the sEPSCs frequency significantly (106.7 ± 7.9%, n = 10, Fig 3C), when compared to the SB 366791 pre-treatment period. These results suggest that SLIGKV-NH_2_ induced increase of sEPSC frequency through activation of spinal TRPV1 receptors.

Staurosporine (250 nM, 4 min) application had no effect on the sEPSC frequency (90.8 ± 10.1%, n = 9) in the next experiments. Subsequent co-application of staurosporine (250 nM) with SLIGKV-NH_2_ (100 μM, 4 min) prevented any significant change of the sEPSC frequency (108.5 ± 7.2%, n = 9, Fig 3C), compared to the pre-treatment period with staurosporine alone. Inhibition of protein kinases thus counteracted the PAR2 activation-induced excitatory effect on the sEPSC frequency.

The mean value of sEPSCs frequencies recorded after SLIGKV-NH_2_ application alone was significantly different from those recorded in the presence of SLIGKV-NH_2_ with SB 366791 and starosporine (^#^p < 0.05, Fig 3C). These results suggest that the PAR2 induced increase of sEPSC frequency was at least partially mediated by TRPV1 receptors and protein kinases activation.

The average amplitude of the recorded sEPSCs did not change significantly in any of the experimental conditions (control 25.7 ± 2.1 pA, SLIGKV-NH_2_ 24.6 ± 1.8 pA, n = 17; control 25.7 ± 1.2 pA, VKGILS -NH_2_ 25.9 ± 1.1 pA, n = 6; control 24.6 ± 2.9 pA, SB 366791 23.8 ± 1.2 pA, SB 366791/SLIGKV-NH_2_ 23.8 ± 1.4 pA, n = 10; control 24.0 ± 1.9 pA, staurosporine 23.6 ± 2.1 pA, staurosporine/SLIGKV-NH_2_ 24.3 ± 2.1 pA, n = 9). No change of sEPSC amplitude was also detected using cumulative amplitude analysis for the first group of neurons (n = 17, Fig 3B).

### PAR2 mediated modulation of dorsal root stimulation-evoked EPSCs

Modulation of eEPSCs by PAR2 activation was tested in superficial dorsal horn neurons, where dorsal root attached to the spinal cord slice was electrically stimulated with a glass suction electrode in 30 s intervals. Evoked EPSCs were recorded in 41 neurons and 37 of these showed an increase of sEPSC frequency (5.6 ± 0.7 Hz, n = 37, p < 0.001) after capsaicin (0.2 μM) application at the end of the experiment compared to frequency of sEPSC (1.6 ± 0.1 Hz, n = 41) recorded under control condition.

**Fig 4:**
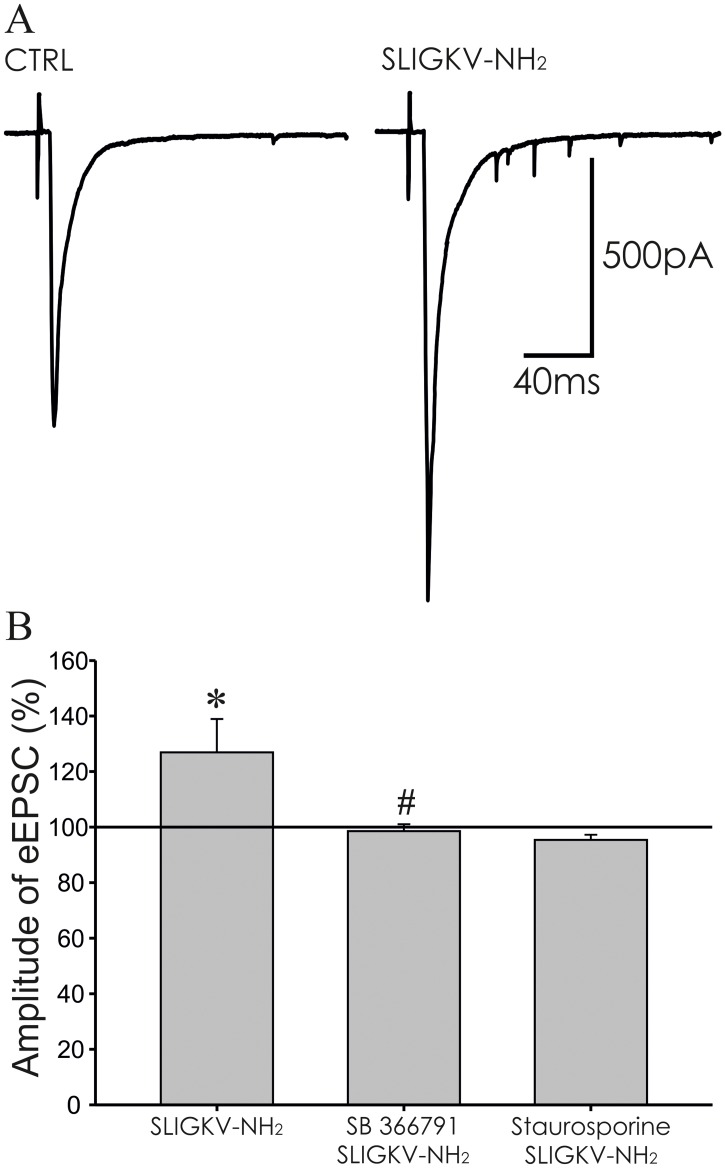
Activation of PAR2 increased the amplitude of EPSCs evoked by dorsal root stimulation. **(A)** Application of SLIGKV-NH_2_ (100 μM, 4 min) increased the amplitude of the evoked EPSC. **(B)** The increase of eEPSCs amplitude during the SLIGKV-NH_2_ (100 μM, 4 min) application was statistically significant compared to pre-treatment values (n = 17, *p < 0.05). Application of SB 366791 (10 μM, 4 min, n = 10) or staurosporine (250 nM, 4 min, n = 9) prevented the SLIGKV-NH_2_ induced eEPSC amplitude increase. The mean eEPSC frequency of SB 366791 and SLIGKV-NH_2_ co-application was statistically different from the application of SLIGKV-NH_2_ alone (^#^p < 0.05).

In the first series of experiments bath application of SLIGKV-NH_2_ (100 μM, 4 min) increased the amplitude of evoked EPSCs (126.9 ± 12.0%, n = 17, p < 0.05, Fig 4A and 4B). The increase of the eEPSC amplitude was even higher during the 4 min period following the SLIGKV-NH_2_ application (washout period; 148.9 ± 17.7%, p < 0.01). Application of the control inactive peptide (VKGILS-NH_2_, 100 μM, 4 min) did not change the eEPSC amplitude (98.79 ± 12.22%, n = 6). These results suggest that activation of PAR2 may enhance synaptic transmission in the superficial spinal cord dorsal horn.

Application of SB 366791 (10 μM, 4 min) itself had no effect on the amplitude of the eEPSCs (105.5 ± 4.4%, n = 10). Subsequent co-application of SB 366791 (10 μM) with SLIGKV-NH_2_ (100 μM, 4 min) did not lead to eEPSC amplitude change during application (98.5 ± 2.5%, Fig 4B) and during the washout period (101.8 ± 6.8%). Inhibition of spinal TRPV1 receptors thus prevented the PAR2 activation-induced increase of eEPSC amplitude.

In another group of neurons staurosporine (250 nM, 4 min) application did not change the amplitude of eEPSCs (101.8 ± 4.7%, n = 9). This pretreatment and subsequent co-application of SLIGKV-NH_2_ (100 μM, 4 min) with staurosporine (250 nM) had no effect on the eEPSC amplitude (96.9 ± 3.4%, Fig 4B) during application and during the washout period (91.7 ± 4.8%). Inhibition of PKs thus prevented the increase of eEPSC amplitude induced by PAR2 activation.

## Discussion

The important role of PAR2 in nociception was demonstrated in a variety of pathological pain conditions [4,18,37,48–50]. However, the modulation of excitatory synaptic transmission in the spinal cord superficial dorsal horn by PAR2 was studied only marginally with various results [15,16]. In this work, we have further studied the role of spinal PAR2 activation on modulation of nociceptive synaptic transmission. In our *in vivo* experiments the intrathecal application of PAR2 activating peptide SLIGKV-NH_2_ induced thermal hyperalgesia in naive adult rats that was prevented by inhibition of spinal TRPV1 receptors and attenuated by inhibition of protein kinases. However, sensitivity to mechanical stimuli did not change in the same experiments. Recordings of mEPSCs from neurons in lamina I and II_(outer)_ in spinal cord slices *in vitro* revealed robust decrease of their frequency after bath application of SLIGKV-NH_2_. The same SLIGKV-NH_2_ treatment elicited an increase of sEPSCs frequency and amplitude of dorsal root stimulation-evoked EPSCs. All these effects on EPSC *in vitro* were attenuated by antagonists of TRPV1 receptors and protein kinases.

Our results indicated presence of several hours lasting thermal hyperalgesia after intrathecal administration of PAR2 activating peptide SLIGKV-NH_2_, which corresponds to the earlier findings [15]. However, this treatment failed to induce mechanical allodynia, which was previously shown after intrathecal application of another PAR2 activating peptide SLIGRL-NH_2_ [15,17]. This activating peptide was formerly considered as a specific PAR2 agonist, but recently activation of several Mrg (Mas-related G-protein-coupled) receptors that induce itch in mice was demonstrated [51,52]. Nevertheless SLIGRL-NH_2_ induced mechanical hypersensitivity was absent in PAR2 knock-out mice (Alier et al., 2008), pointing probably to different experimental approaches and conditions and/or distinctive mechanisms in different animal species, than to the specificity of these two PAR2 activating peptides. Our results indicate that under control conditions, activation of spinal PAR2 leads preferentially to thermal hypersensitivity. This may change under pathological conditions, like bone cancer-evoked pain, when PAR2 are overexpressed predominantly in medium and large DRG neurons [36], which could underlie the development of mechanical hypersensitivity. Thermal hyperalgesia induced in our experiments by activation of spinal PAR2 was prevented by inhibition of spinal TRPV1 receptors. In naïve animals the thresholds for thermal and tactile stimuli in the peripheral nerves endings should be unchanged, when PAR2 activating peptide is injected intrathecally. It seems plausible to suggest that modulation at the spinal cord level of the incoming action potentials generated in the periphery by the thermal stimulus applied on the paw induced the observed thermal hyperalgesia. This hyperalgesia was thus most likely mediated by changes in presynaptic endings co-expressing PAR2 and TRPV1 receptors. It is possible that activity induced by the mechanical stimuli was conducted by primary afferents that did not co-express these receptors and thus PAR2 agonist application did not change their synaptic transmission and did not evoke increased mechanical sensitivity.

The mechanism involving TRPV1 activation in PAR2-induced hyperalgesia was demonstrated also after the activation of peripherally localized PAR2 [13] and this corresponds well to TRPV1 mediated thermal hypersensitivity [53]. If spinal TRPV1 were sensitized after PAR2 activation, it is plausible that body temperature and/or endogenous ligands subsequently activated TRPV1. In addition it was demonstrated that activation of PAR2 reduced the temperature threshold required for TRPV1 activation to the body temperature in cultured cells [14]. In our experiments intrathecal administration of staurosporine, a broad spectrum PKs inhibitor (with the highest affinity for PKC), partially attenuated the thermal hyperalgesia induced by spinal PAR2 activation. This suggests the involvement of PKC in the process, most likely through phosphorylation of TRPV1 receptors [26,33,34]. Attenuation of spinal inhibitory synaptic transmission by PAR2, demonstrated by reduced frequency and amplitude of sIPSCs in the spinal cord dorsal horn [17], may also contribute to the hypersensitivity development.

The potential underlying mechanisms of the behavioural changes were studied *in vitro*. In our experiments, the frequency of sEPSCs and amplitude of the dorsal root stimulation-evoked eEPSC were increased after PAR2 activating peptide (SLIGKV-NH_2_) application. Similar increase of sEPSCs frequency induced with the same peptide (SLIGKV-NH_2_) application was reported before in experiments with low concentration applications (3 μM and 5 μM) [16]. In contrast, bath application of other PAR2 activating peptide SLIGRL-NH_2_ (10 μM) had no significant effect on the sEPSC frequency in lamina II neurons [15].

We have newly demonstrated that application of PAR2 activating peptide increased the amplitude of evoked EPSCs and this effect was blocked by TRPV1 antagonist SB 366791. In addition the same mechanism was present in the PAR2-induced increase of sEPSCs frequency in our experiments. The sensitization of TRPV1 receptors by PAR2 activation was shown previously in DRG neurons [13]. PAR2-induced effects on EPSCs in our recordings were mediated also by PKs in accordance with finding that PAR2 stimulation leads to TRPV1 sensitization via PKCε and PKA [34]. Under our *in vitro* conditions with room temperature experiments it is more likely that endogenous substances may have activated spinal TRPV1 receptors. It was demonstrated before, that a low concentration of lipophilic endogenous ligand (N-oleoyldopamine, OLDA) activated sensitized TRPV1 receptors in spinal cord slices under similar conditions [35]. We cannot exclude the possibility that the dependence of TRPV1 activation on membrane voltage could also play a role in the process [54]. In addition, PAR2 activation leads to enhanced release of pro-nociceptive peptides (SP, CGRP) from central endings of DRG neurons [11,13] that may further modulate synaptic transmission and enhance nociceptive output from the spinal cord to the brain. The increase of sEPSC frequency by PAR2 activation could involve also mobilization of Ca^2+^ from intracellular stores and increased Ca^2+^ influx through other ion channels [19,55,56].

In the series of our experiments where TTX was present in the extracellular solution, PAR2 activation induced decrease of the mEPSCs frequency. Surprisingly, this decrease was also largely dependent on the TRPV1 receptor activation, while in other experiments TRPV1 receptors activation lead to increase of mEPSC frequency [35,46]. These results indicate that under conditions, when TTX-sensitive sodium channels are blocked, another presynaptic mechanism induced by PAR2 activation predominated and resulted in decrease of glutamate release from the central endings of DRG neurons expressing also TRPV1 receptors. This observation could be explained by functional and physical connection between TRPV1 and large-conductance calcium- and voltage-activated potassium (BK) channels [57]. On DRG neurons, TRPV1 and BK channels form complex, which could allow the activation of BK channels by increased local concentration of Ca^2+^ ions through TRPV1 [57]. Due to outflow of K^+^ ions from the cell through the BK channels, when TTX-sensitive Na^+^ channels are blocked, the hyperpolarization could occur and the release of glutamate could be reduced. Another plausible mechanism could be the inhibition of voltage activated Ca^2+^ channels by TRPV1 activation. Olvanil, a non-pungent TRPV1 agonist, profoundly inhibited (approximately 60%) N-, P/Q-, L-, and R-type voltage-activated Ca^2+^ channel current in DRG neurons [58]. The effect induced by olvanil was dependent on calmodulin and calcineurin activity. However, the mechanisms participating in TRPV1 activation and the subsequent intracellular responses may differ according to agonist used and receptor subtype [59]. Recently, it was demonstrated that stochastic opening of voltage-activated Ca^2+^ channels is a major trigger for miniature glutamate release in hippocampal synapses [60]. This finding supports the possible occurrence of decreased glutamate release from presynaptic endings of DRG neurons induced by PAR2 activation and mediated by TRPV1 modulation of voltage-activated Ca^2+^ channels in our conditions, when mEPSCs are recorded in acute spinal cord slices. Nevertheless, these two hypotheses require further investigation.

Miniature and spontaneous EPSCs may be both recorded in superficial dorsal horn neurons spontaneously, without any stimulation. In our preparations, potential self-generated formation and propagation of action potentials was prevented by blocking sodium channels with TTX during the recording of mEPSC. It was suggested that mEPSCs reflect only the spontaneous release of readily releasable pool of synaptic vesicles. However, it is not clear what modulatory changes may induce decrease or increase of mEPSCs frequency in synaptic transmission of superficial spinal cord dorsal horn neurons. In comparison the PAR2-induced effect on the sEPSCs and eEPSCs probably reflect more closely the mechanisms involved in the observed behavioural changes. To elucidate the PAR2-induced reduction of the mEPSCs frequency will require further experiments.

Our results imply that PAR2 receptors may play an important role in nociceptive synaptic transmission at the spinal cord level. This PAR2-induced modulation of nociception is at least partially dependent on TRPV1 receptors activation. It seems plausible to suggest that their role may be potentiated during pathological processes, when expression of both PAR2 and TRPV1 receptors is enhanced [61–63].
